# Supplementary material for: Towards synthetic cells using peptide-based reaction compartments
Source: Nat Commun. 2018 Sep 21;9:3862. doi: 10.1038/s41467-018-06379-8 (PMC6154970; doi:10.1038/s41467-018-06379-8)
Supplement: Supplementary file 1 — Supplementary Information [file 41467_2018_6379_MOESM1_ESM.pdf]

Supplementary Information:

Towards synthetic cells using peptide-based reaction compartments

Kilian Vogele[†], Thomas Frank[†], Lukas Gasser[†], Marisa A. Goetzfried[†], Mathias W. Hackl[§], Stephan A. Sieber[§], Friedrich C. Simmel[†,⊥], and Tobias Pirzer[†*]

[†]Physics of Synthetic Biological Systems - E14, Physics-Department and ZNN, Technische Universität München, 85748 Garching, Germany
[§]Department of Chemistry, Center for Integrated Protein Science Munich (CIPSM), Technische Universität München, Lichtenbergstraße 4, 85748 Garching, Germany
[⊥]Nanosystems Initiative Munich, 80539 Munich, Germany

**Contact:* pirzer@tum.de

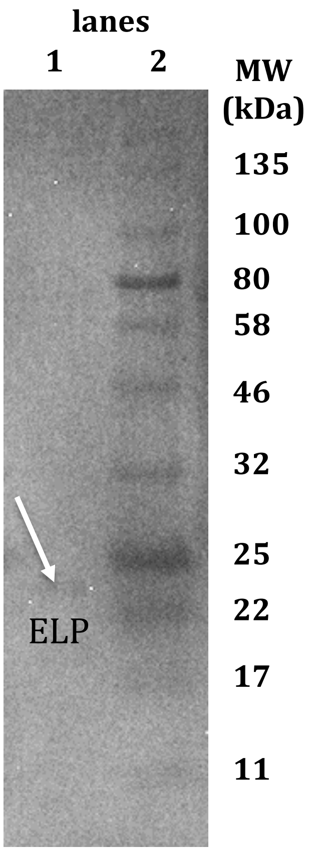

Supplementary Figure 1 | SDS gel of purified EF. Lane 1 shows the purified EF peptide. Lane 2 is the Color Prestained Protein Standard ladder, Broad Range (11–245 kDa) from NEB. EF has a mass of about 18 kDa, but the peptide band is shifted upwards to about 23 kDa, which is a well-known effect for elastin-like peptides (see main part).

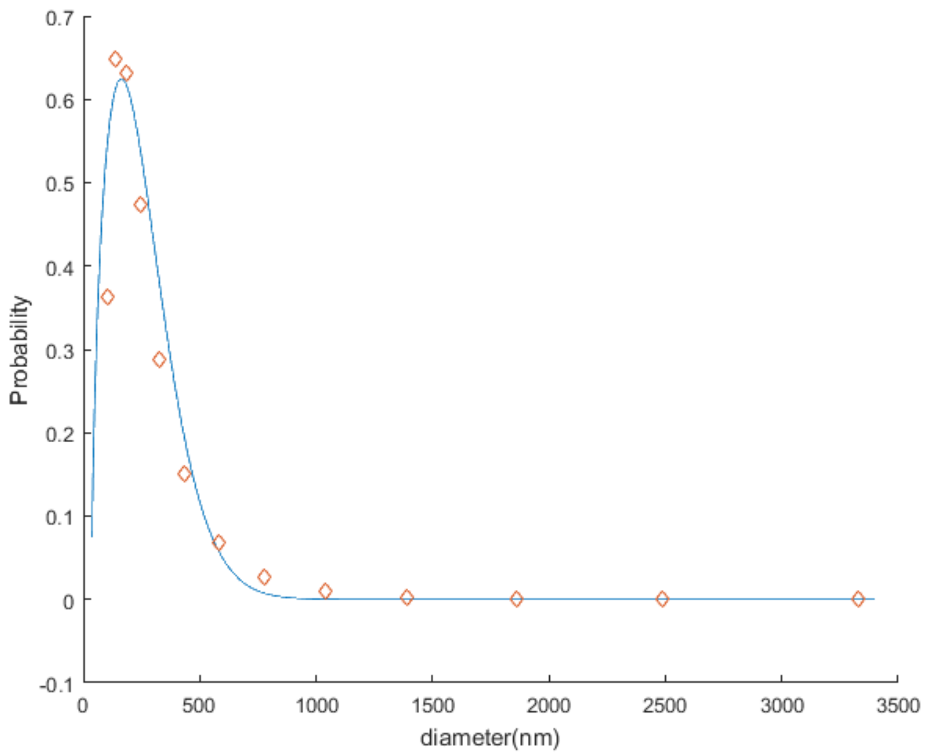

Supplementary Figure 2 | Appearance probability of the vesicles measured with DLS. The vesicles were produced using the glass beads method, 180 pM EF and 1x PBS as the swelling solution. Original DLS data from a binned histogram (red diamonds) is shown with a peak value of 178 nm and a dispersion of 67 nm. The blue solid curve is a Weibull probability distribution fit.

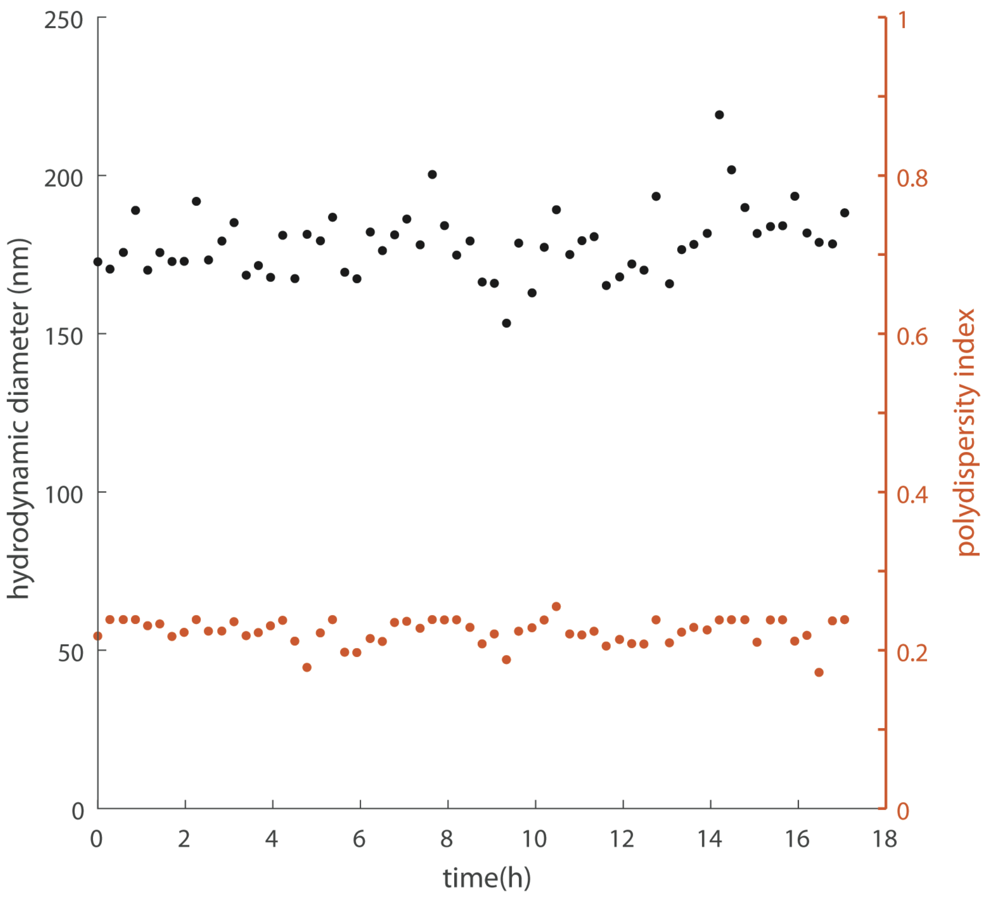

Supplementary Figure 3 | Size stability in time. Measurement of the hydrodynamic diameter (black dots) and the polydispersity index (red dots) of the vesicles for 18 h using DLS. The vesicles were produced using the glass beads method and 1x PBS as the swelling solution. The mean of the diameter is 179 nm with a standard deviation of 10 nm.

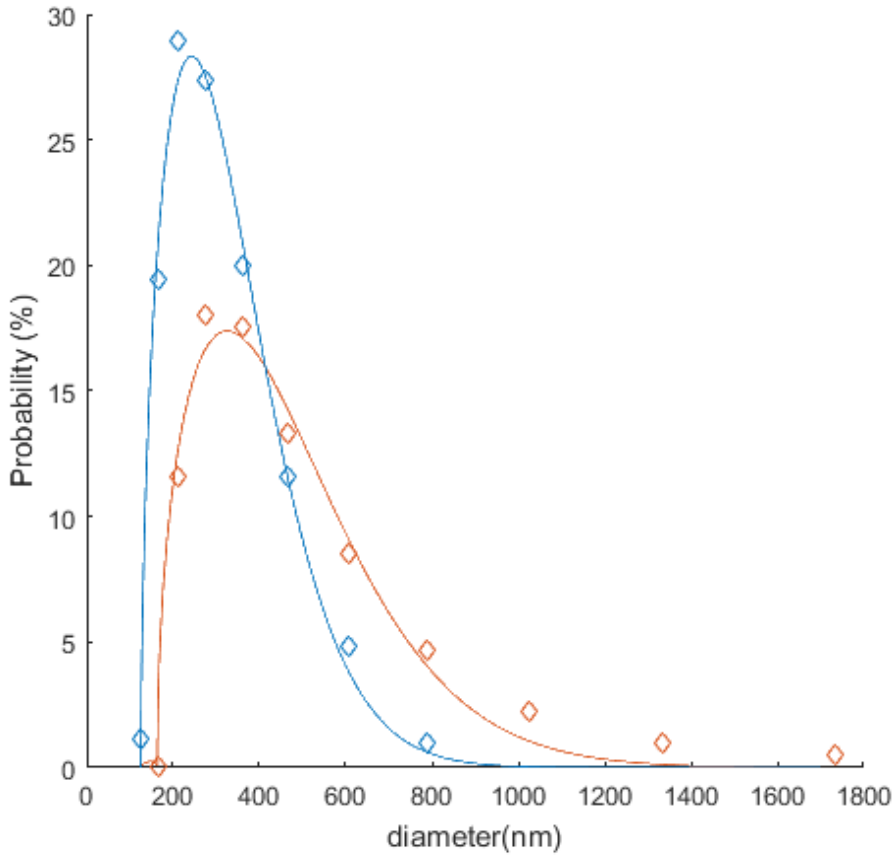

Supplementary Figure 4 | Vesicle growth through the addition of EF from the outside. Appearance probability of the vesicles measured with DLS. The vesicles were produced using the glass beads method and 1x PBS as the swelling solution. The blue diamonds are original DLS data from a binned histogram. The solid blue cure is a Weibull probability distribution fit. The peak value of the diameter was found to be 192 nm with a dispersion of 74 nm. After the addition of 50 µM EF the peak value of the binned histogram (red diamonds) changed to 234 nm with a dispersion of 101 nm.

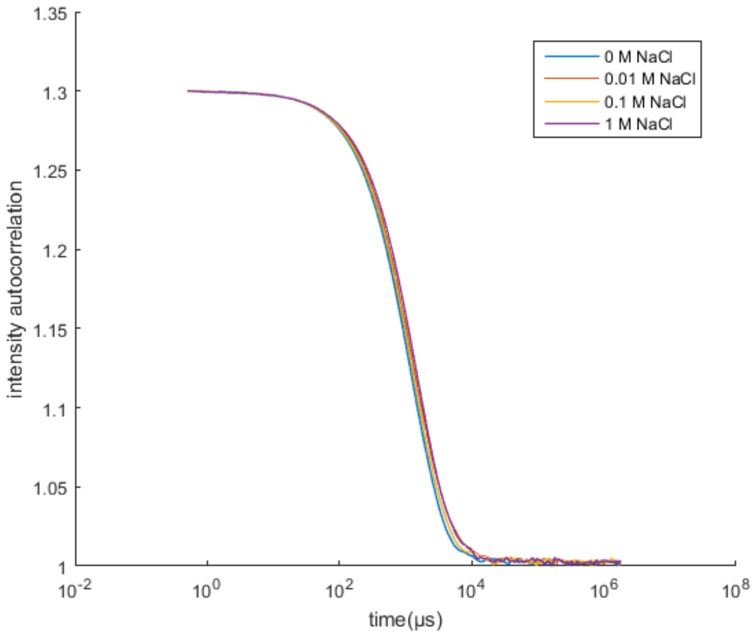

Supplementary Figure 5 | Membrane stability in NaCl gradients. After vesicle formation using the glass beads method and 1x PBS we tested the vesicles size stability for NaCl concentration gradients between outside and inside. Therefore, we added NaCl to outside solution to get NaCl concentration differences of 0 M, 0.01 M, 0.1 M and 1 M. The correlation functions from DLS measurements show no variations for these NaCl concentrations. In TEM measurements we see spherical vesicels for all gradients except 1 M NaCl with some vesicles showing ellipsoid shapes.

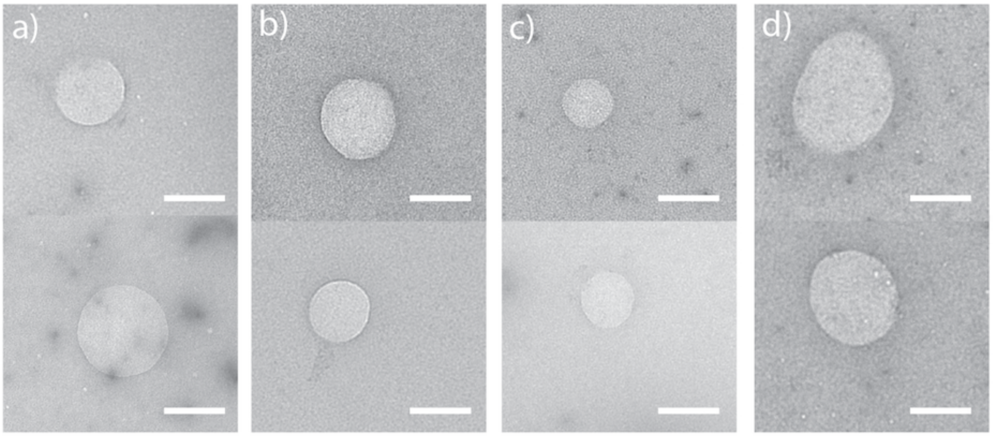

Supplementary Figure 6 | Shape of EF vesicles at various NaCl membrane gradients. The concentration difference of NaCl between inside and outside the vesicles are: 0 M (blue), 0.01 M (red), 0.1 M (yellow) and 1 M (purple). Scale bar, 200 nm.

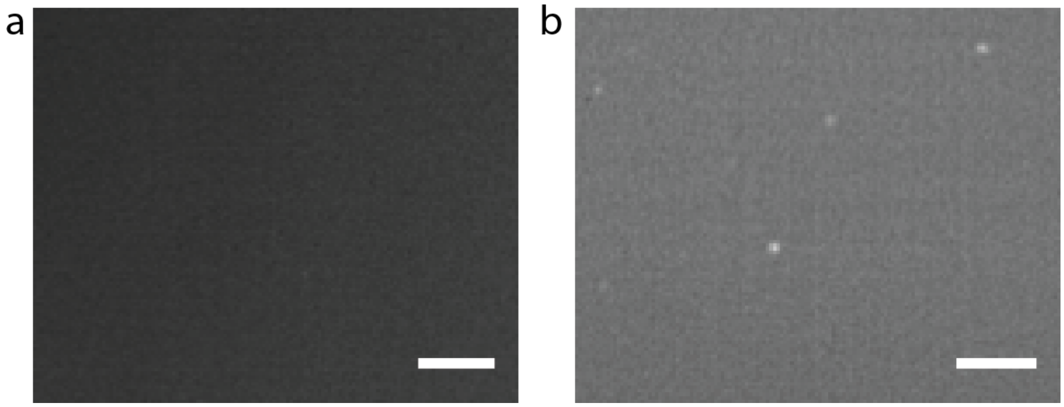

Supplementary Figure 7 | Light microscopy of vesicles with encapsulated fluorophore-labeled DNA. As described in the main part EF vesicles were produced with encapsulated fluorophore-labeled DNA with 1x PBS. **a** Brightfield image of EF vesicles. Due to their size of about 200 nm and the diffraction limit there are no vesicles visible. **b** Fluorescence microscopy image of the same vesicles. Scale bar, 10 µm.

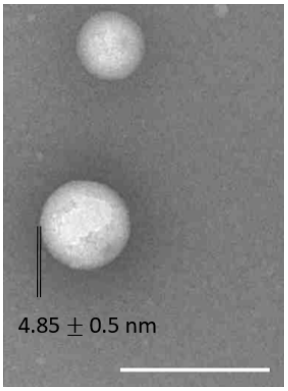

Supplementary Figure 8 | Determination of membrane thickness. TEM image of EF vesicles used for the determination of the membrane thickness. The glass beads where rehydrated with 1x PBS. The thickness of the membrane was calculated for these vesicles to 4.9 nm with a standard deviation of 0.5 nm. Scale bar: 200 nm.

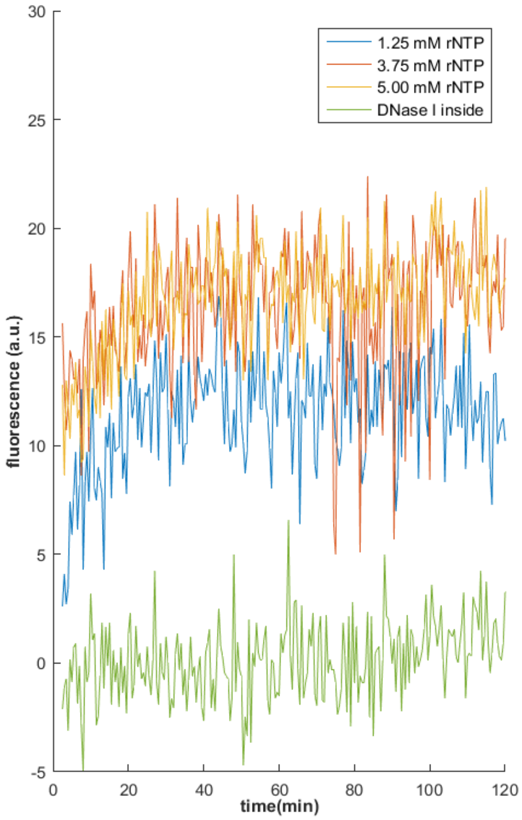

Supplementary Figure 9 | TX in vesicles: transcription of the dBroccoli aptamer. Fluorescence time traces during the transcription of the dBroccoli aptamer inside the EF vesicles. Here, only transcription mixture TX was encapsulated using the glass beads method. After vesicle formation DNase I was added to prevent transcription and hence fluorescence at the outside. Excitation was carried out at 480 nm and the emission was measured at 520 nm. Three different rNTP concentrations were used, namely 1.25 mM (blue), 3.75 mM (red) and 5.00 mM (yellow). As a negative control DNase I was also incorporated into the vesicles with 1.25 mM rNTP (green). For signal correction a sample containing vesicles with transcription mixture TX but without DNA template was used.

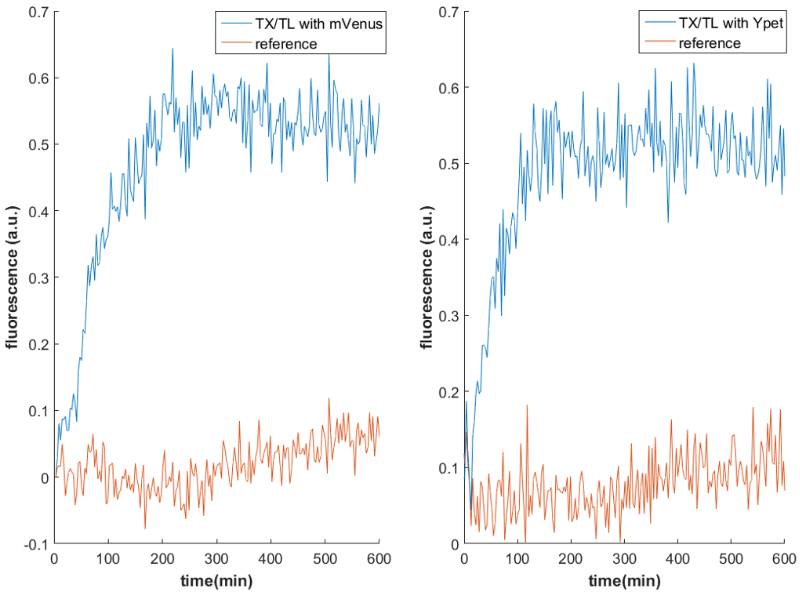

Supplementary Figure 10 | *In vesicular* translation vs. time. Fluorescence signals (excitation at 500 nm and emission at 520 nm) of two fluorescent proteins (blue curves), namely mVenus (left) and Ypet (right), which are expressed inside of EF vesicles utilizing TX-TL. For mVenus expression a T7 promoter was used and for Ypet expression a constitutive promoter from E. coli was used. To suppress expression at the outside an antibiotic was added to the solution surrounding the vesicles. For a reference the antibiotic was also encapsulated inside the vesicles and successfully suppressed protein expression (red curves). Both signals (blue and red) were background corrected using a sample containing EF vesicles with TX-TL, but no plasmids.

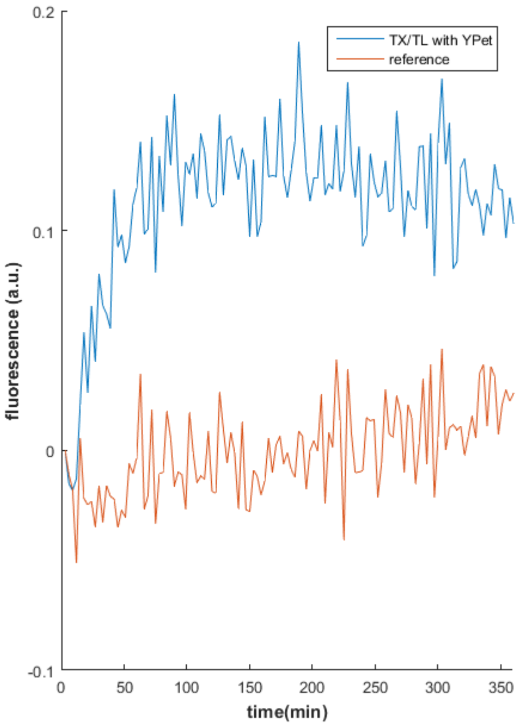

Supplementary Figure 11 | YPet expression inside vesicles. Fluorescence signals (excitation at 500 nm and emission at 520 nm) of YPet (blue curves), namely, which are expressed inside of EF vesicles utilizing TX-TL. For Ypet expression a constitutive promoter from E. coli was used. To suppress expression at the outside EDTA was added to the solution surrounding the vesicles. For a reference EDTA was also encapsulated inside the vesicles and successfully suppressed protein expression (red curves). Both signals (blue and red) were background corrected using a sample containing EF vesicles with TX-TL, but no plasmids.

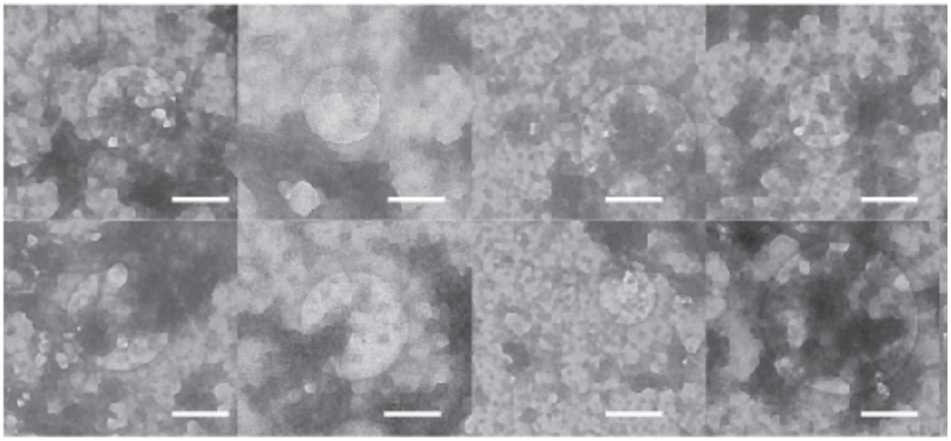

Supplementary Figure 12 | EF expression in vesicles using TX-TL. TEM images of the EF vesicles after 0 min incubation time. The TX-TL system and the plasmid encoding EF are encapsulated with the glass beads method. After vesicle formation an antibiotic is added to the outer solution. The samples were not purified from the TX-TL (see main text). All shown subfigures are from the same TEM grid and sample. Scale bars: 200 nm.

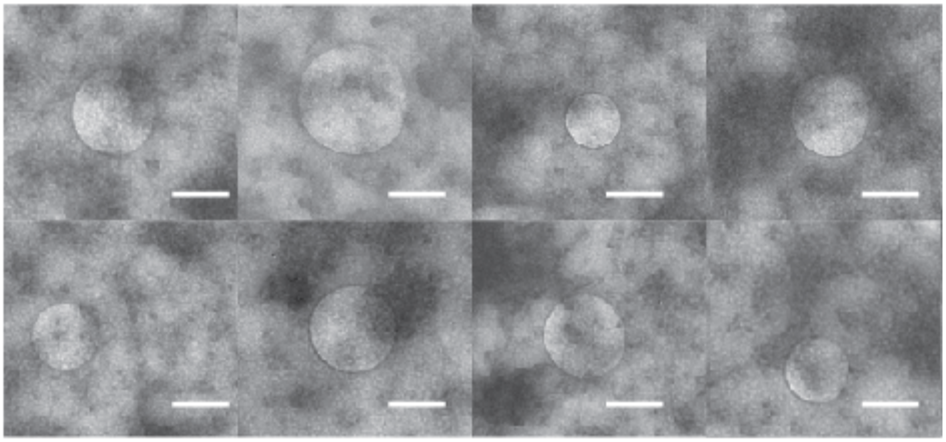

Supplementary Figure 13 | EF expression in vesicles using TX-TL. TEM images of the EF vesicles after 240 min incubation at 29 °C. The TX-TL system and the plasmid encoding EF are encapsulated with the glass beads method. After vesicle formation an antibiotic is added to the outer solution. The samples were not purified from the TX-TL (see main text). All shown subfigures are from the same TEM grid and sample. Scale bars: 200 nm.

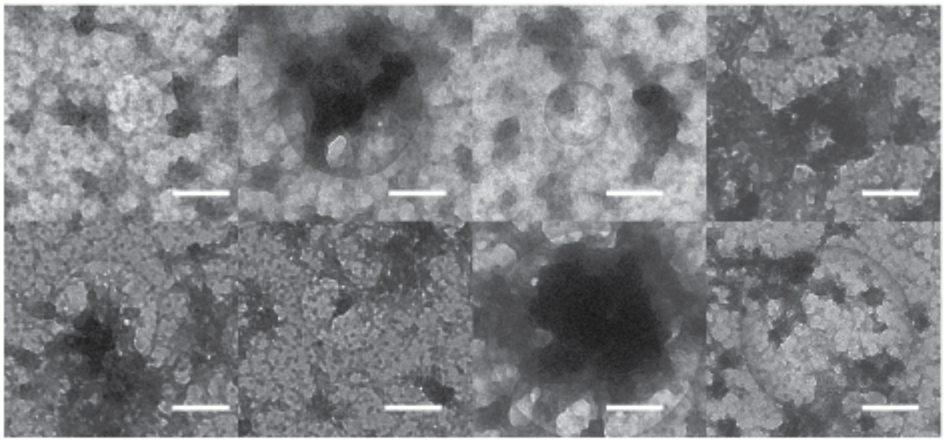

Supplementary Figure 14 | V40 expression in vesicles using TX-TL. TEM images of the EF vesicles after 0 min. The TX-TL system and the plasmid encoding V40 are encapsulated with the glass beads method. After vesicle formation an antibiotic is added to the outer solution. The samples were not purified from the TX-TL (see main text). All shown subfigures are from the same TEM grid and sample. Scale bars: 200 nm.

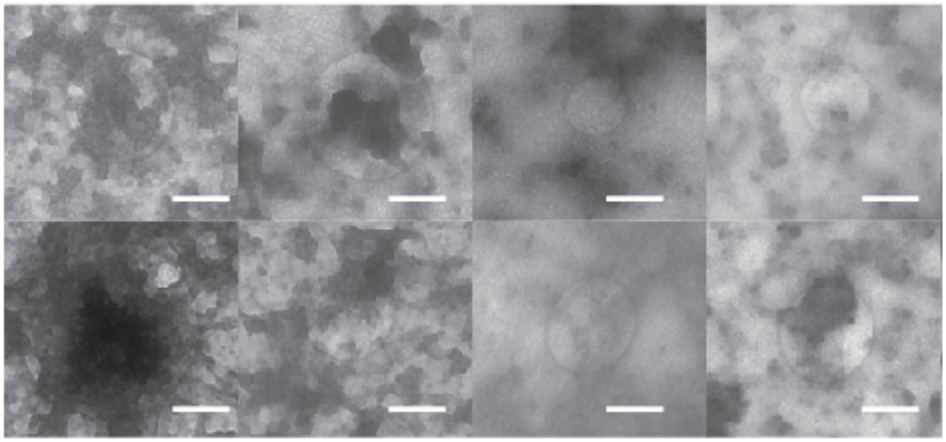

Supplementary Figure 15 | V40 expression in vesicles using TX-TL. TEM images of the EF vesicles after 240 min incubation at 29°C. The TX-TL system and the plasmid encoding V40 are encapsulated with the glass beads method. After vesicle formation an antibiotic is added to the outer solution. The samples were not purified from the TX-TL (see main text). All shown subfigures are from the same TEM grid and sample. Scale bars: 200 nm.

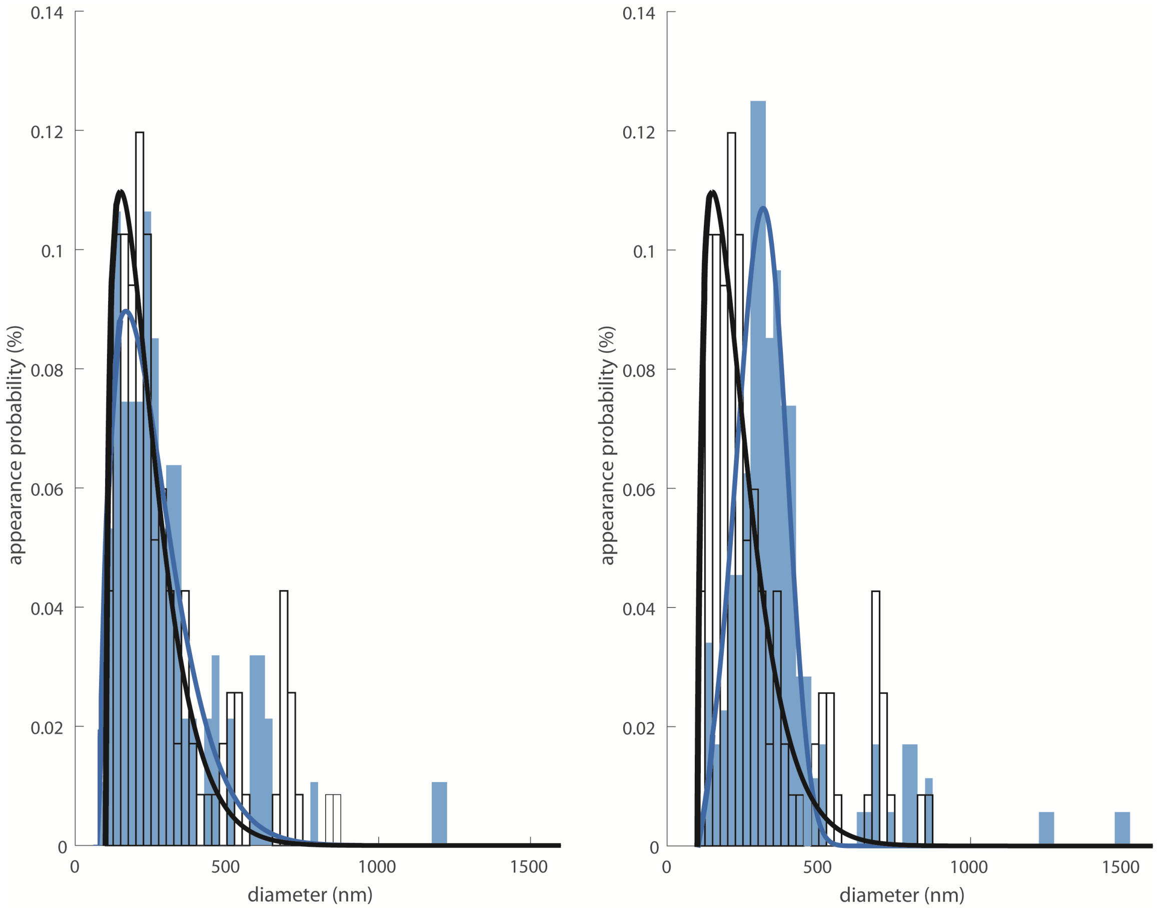

Supplementary Figure 16 | Vesicle size. Size distributions of the vesicles measured by TEM, from about 100 vesicles. (Left) Vesicles containing TX-TL and the V40 plasmid. The black framed histogram shows the size distribution at $t = 0$ min. The solid black line is a Weibull function fitted to the histogram resulting in a peak value of 149 nm. The blue histogram is the size distribution at $t = 240$ min. The solid blue line is a Weibull function fitted to the histogram resulting in a peak value of 145 nm. (Right) Vesicles containing TX-TL and the EF plasmid. The black framed histogram shows the size distribution at $t = 0$ min. The solid black line is a Weibull function fitted to the histogram resulting in a peak value of 157 nm. The blue histogram is the size distribution at $t = 240$ min. The solid blue line is a Weibull function fitted to the histogram resulting in a peak value of 330 nm. For the statistical analysis about 100 vesicles were evaluated.

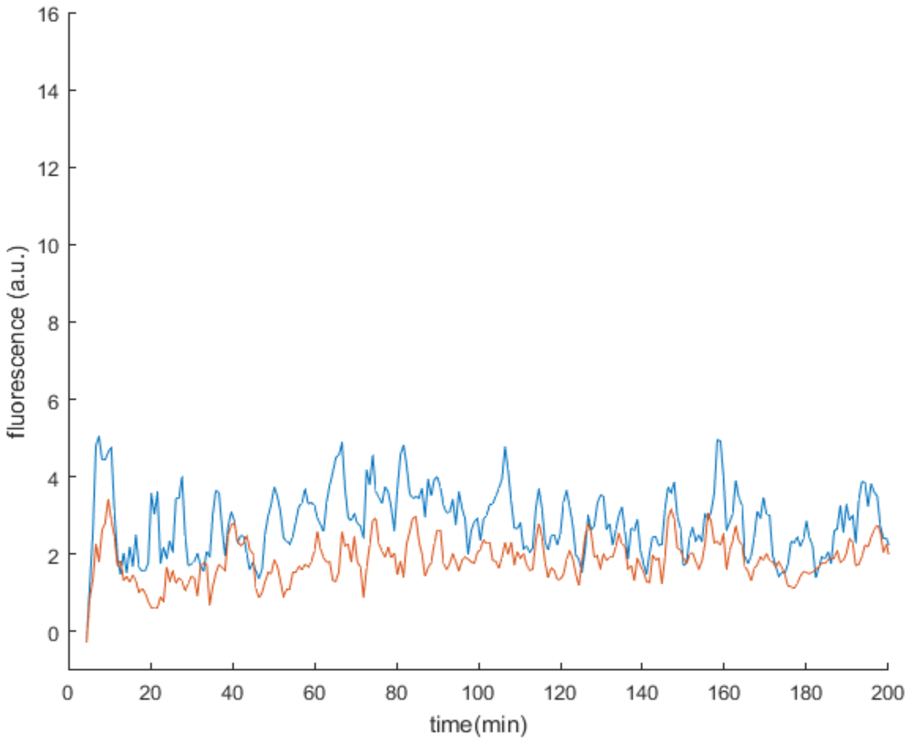

Supplementary Figure 17 | Negative control of the FRET assay. Time trace of the fluorescence signal of the donor dye Cy3 (red) and the acceptor dye Cy5 (blue), which are attached to EF via copper-based azide-alkyne Huisgen cycloaddition. The TX-TL mixture, the EF plasmid and kanamycin were encapsulated in vesicles made of Cy5-EF and Cy3-EF using the glass beads method. Both signals are background corrected with a sample containing vesicles made of Cy5-EF and Cy3-EF, but without TX-TL and plasmid.

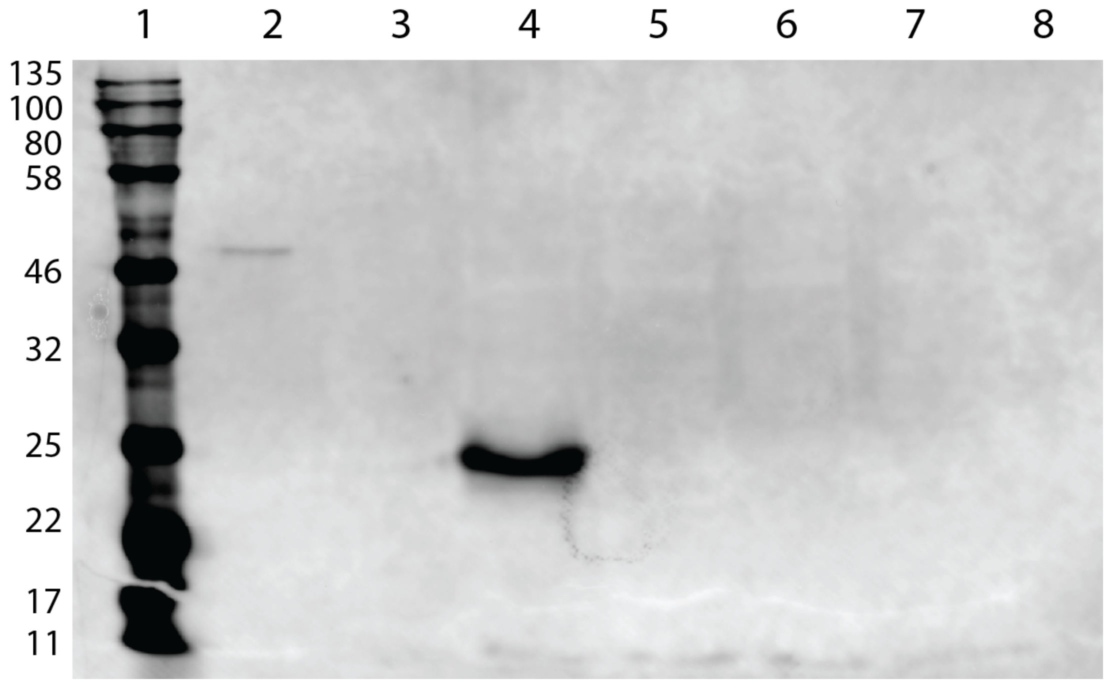

Supplementary Figure 18 | Western blot of the peptides EF-His (6x histidin) expressed inside the vesicles. The first lane (from left) is the Color Prestained Protein Standard ladder, Broad Range (11–245 kDa) from NEB. The second lane is a positive control with a 67 kDa histidine-tagged helicase. For lane three a sample with vesicles containing TX-TL and the EF-His plasmid without incubation was used. For lane four a sample with vesicles containing TX-TL and the EF-His after an incubation time of 240 min at 29 °C was used. The sample for lane five is the same as for lane three, but with kanamycin inside the vesicles. The sample for lane six is the same as for lane four, but also with kanamycin inside the vesicles. For lane seven only TX-TL without any ELP was used. For lane eight a sample with peptide vesicles in 1x PBS was used. The used antibodies were purchased from Life Technologies GmbH, Darmstadt, Germany. Primary antibody: anti-His antibody (6x-His Epitope tag antibody) (mouse, Lot: SI252938, Catalogue: MA1135). Secondary antibody: anti-mouse Alexa Fluor 680 (goat, Lot: RG233737A, Catalogue: A28183).

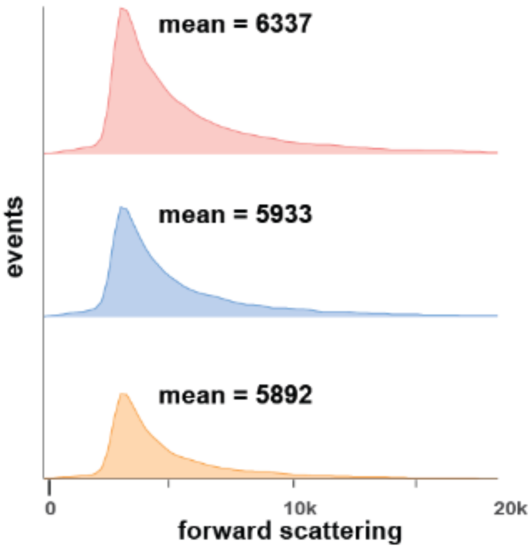

Supplementary Figure 19 | Forward scattering of the EF vesicles using flow cytometry. (Top) The red histogram shows the scattering of vesicles containing TX-TL and the EF plasmid for peptide expression. The sample was incubated for eight hours at 29 °C. To suppress expression outside the vesicles the antibiotic kanamycin was added to the external solution. The mean of the forward scattering was measured to be 6337, which indicates vesicle growth. (Center) The blue histogram shows the scattering of vesicles containing TX-TL and the V40 plasmid. It was also incubated for eight hours at 29 °C. (Bottom) The yellow histogram shows the scattering of vesicles containing TX-TL, the EF plasmid and the antibiotic kanamycin to suppress peptide expression.

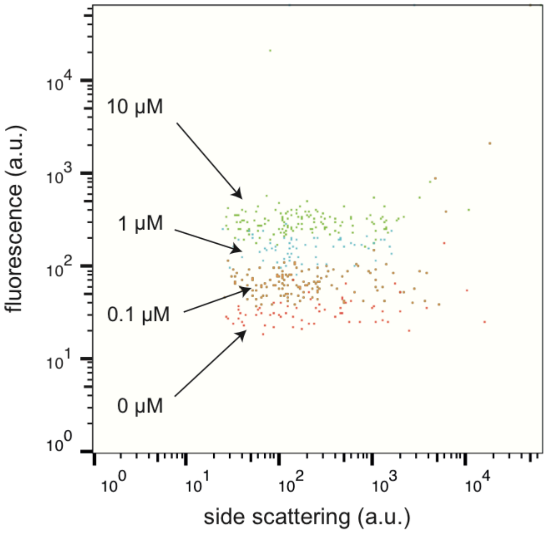

Supplementary Figure 20 | Flow Cytometry reference. Flow cytometry measurement of the FAM-labeled DNA without EF with starting concentrations of 10 µM (green dots, 1.7 µM after dilution for the measurement), 1 µM (blue dots, 0.17 µM after dilution), 0.1 µM (orange dots, 0.02 µM after dilution) and the control without dyes (red dots).

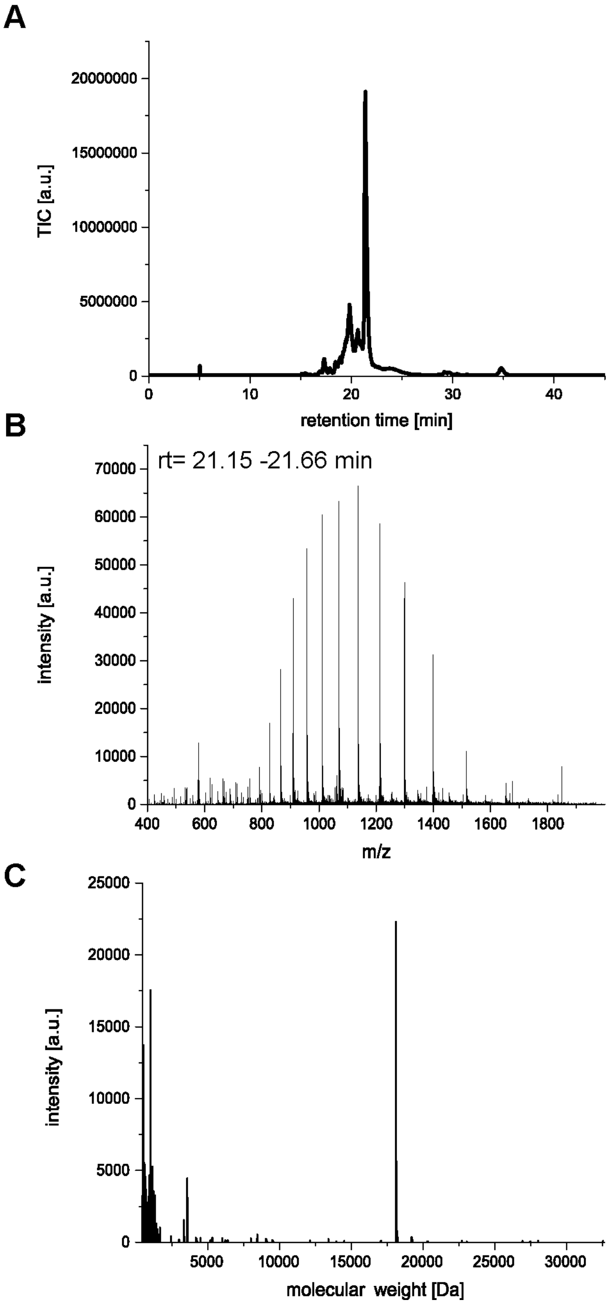

Supplementary Figure 21 | Intact-protein mass spectrometry of EF. (A) Total ion current chromatogram. (B) Summed-up raw spectra of retention time 21.15 to 21.66 min. (C) Deconvoluted spectra of retention time 21.15 to 21.66 min. EF is the only prominent species.

Supplementary Note 1. Weibull distribution

To obtain parameters of the size distributions a Weibull distribution function was used with the following probability density function

$$f(d) = \frac{\delta}{\eta} \cdot \left(\frac{d - d_0}{\eta}\right)^{\delta - 1} \cdot \exp\left[-\left(\frac{d - d_0}{\eta}\right)^{\delta}\right] \tag{1}$$

with d as the diameter of the vesicles and η, δ and d_0 as free parameters. The peak value where the distribution of the diameter reaches its maximum can be obtained with the equation:

$$d_{max} = d_0 + \eta \left(\frac{\delta - 1}{\delta}\right)^{\frac{1}{\delta}} \tag{2}$$

The expected value E can be calculated using

$$E = d_0 + \eta \cdot \Gamma\left(\frac{1}{\delta} + 1\right) \tag{3}$$

and the dispersion (standard deviation) D of the Weibull distribution is calculated through

$$D^2 = \eta^2 \left(\Gamma\left(\frac{2}{\delta} + 1\right) - \Gamma^2\left(\frac{1}{\delta} + 1\right)\right) \tag{4}$$

Supplementary Note 2. Encapsulation efficiency of TX-TL components

The low signal-to-noise ratio of the mVenus and YPet fluorescence signals in Fig. 2 resp. Supplemetary Fig. 6 can be rationalized by the small size of the vesicles resp. the encapsulation efficiency. We assume a Poisson distribution to derive the probability that at least one molecule of a kind is encapsulated inside of each vesicle. The probability that all of the N necessary components of the TX-TL system were encapsulated in a vesicle with volume V and radius R is therefore given by

$$P(R) = \prod_{n=1}^{N} \left[1 - e^{-C_n \cdot V(R)}\right] \qquad (5)$$

where C_n is the concentration (number density) of molecule n that is encapsulated. If we assume that the vesicles have a radius of $R = 150$ nm the concentration of the molecules needed to be in the low µM range to have an encapsulation probability close to 100%, which is only valid for the proteins (µM) and the amino acids (mM). The plasmid, however, had a concentration of 50 nM and therefore had an encapsulation probability of only 35%, resulting in a relatively low and noise fluorescence signal.

Supplementary Note 3. Number of peptides expressed inside the vesicles

To estimate the relative number of peptides incorporated into the membrane, we used the following assumption:

$$\frac{N_{t=240\ min} \cdot V_{ELP}}{N_{t=0\ min} \cdot V_{ELP}} = \frac{V_{vesicle\ shell\ at\ t=240\ min}}{V_{vesicle\ shell\ at\ t=0\ min}} = \frac{\frac{4}{3}\pi R_{t=240\ min}^3 - \frac{4}{3}\pi(R_{t=240\ min} - a)^3}{\frac{4}{3}\pi R_{t=0min}^3 - \frac{4}{3}\pi(R_{t=0\ min} - a)^3} = \frac{R_{t=240\ min}^3 - (R_{t=240\ min} - a)^3}{R_{t=0\ min}^3 - (R_{t=0\ min} - a)^3} \tag{6}$$

where N_t is the number of ELP incorporated into the membrane at time t and the volume of a single peptide V_{ELP}. R_t is the radius of the vesicles at time t and a is the membrane thickness. We further assumed that the volume of a single peptide stays constant and is independent of the vesicle radius, which is supported by the measurement of the membrane thickness, which indicate no swelling of the membrane:

$$\frac{N_{t=240\ min}}{N_{t=0\ min}} = \frac{R_{t=240\ min}^3 - R_{t=240\ min}^3 + 3R_{t=240\ min}^2 a - 3R_{t=240\ min} a^2 + a^3}{R_{t=0\ min}^3 - R_{t=0\ min}^3 + 3R_{t=0\ min}^2 a - 3R_{t=0\ min} a^2 + a^3} = \frac{3R_{t=240\ min}^2 a - 3R_{t=240\ min} a^2 + a^3}{3R_{t=0\ min}^2 a - 3R_{t=0\ min} a^2 + a^3} \tag{7}$$

This would result into a relative membrane volume increase of $\xi = 4.44$ for a membrane thickness a of 5.33 nm, a radius of 157 nm at 0 min and a radius of 330 nm at 240 nm. If we assume that $a^n \ll R^n$ for every n larger than 1 we can simplify the relative volume increase to:

$$\frac{N_{t=240\ min}}{N_{t=0\ min}} \approx \frac{R_{t=240\ min}^2}{R_{t=0\ min}^2} \tag{8}$$

For the same values of the radii we get for the relative membrane volume increase $\xi = 4.42$ which is close to the exact formula.

Supplementary Tables.

EF conc. (pM)	dispersion D (nm)	expected value E (nm)	peak value d_{max} (nm)
**110**	47.0	115.5	86.8
**180**	67.0	207.3	177.9
**220**	110.5	236.3	219.6
**440**	414.6	587.5	249.7

Supplementary Table 1 | Vesicle sizes for various EF concentrations. We used several EF concentrations and tested the concentration influence on the size of the vesicles using the glass beads method and 1x PBS as swelling solution. Supplementary Table 1 shows the different hydrodynamic radii determined from the Weibull fit of the vesicle size distribution, which was obtained by DLS measurements of different EF concentrations. For further experiments we used a concentration of 180 pM, since it worked best with TX-TL.

vesicle content	mean (nm)	Standard deviation (nm)
**1x PBS**	4.9	0.5
**t = 0 min TX-TL EF plasmid**	5.2	0.6
**t = 240 min TX-TL EF plasmid**	5.3	0.7
**t = 0 min TX-TL V40 plasmid**	5.6	0.8
**t = 240 min TX-TL V40 plasmid**	5.7	0.6

Supplementary Table 2 | Membrane thickness of the EF vesicles. The membrane thickness of the EF vesicles was measured by analysing 20 vesicles at a representative spot in the TEM images. For image analysis and processing the Java-based software ImageJ was used. The thickness was determined for five different samples.

vesicle content	variance D^2 (nm^2)	dispersion D (nm)	expected value E (nm)	peak value d_{max} (nm)	number of analyzed vesicles
**1x PBS**	4666	68	213	176	92
**t = 0 min TX-TL EF plasmid**	10866	104	239	157	171
**t = 240 min T-/TL EF plasmid**	6818	83	318	330	173
**t = 0 min TX-TL V40 plasmid**	10505	103	233	150	114
**t = 240 min TX-TL V40 plasmid**	16378	128	247	145	93

Supplementary Table 3 | Weibull curve fitting parameters used in Supplementary Fig. 16.
